# How Molecular Topology Can Help in Amyotrophic Lateral Sclerosis (ALS) Drug Development: A Revolutionary Paradigm for a Merciless Disease

**DOI:** 10.3390/ph15010094

**Published:** 2022-01-14

**Authors:** Maria Galvez-Llompart, Riccardo Zanni, Ramon Garcia-Domenech, Jorge Galvez

**Affiliations:** 1 Instituto de Tecnología Química, UPV-CSIC, Universidad Politécnica de Valencia, 46022 Valencia, Spain; maglllo@itq.upv.es; 2 Molecular Topology and Drug Design Unit, Department of Physical Chemistry, University of Valencia, 46100 Valencia, Spain; riccardo.zanni@uv.es (R.Z.); ramon.garcia@uv.es (R.G.-D.)

**Keywords:** amyotrophic lateral sclerosis, orphan diseases, molecular topology, drug design, QSAR, drug repurposing

## Abstract

Even if amyotrophic lateral sclerosis is still considered an orphan disease to date, its prevalence among the population is growing fast. Despite the efforts made by researchers and pharmaceutical companies, the cryptic information related to the biological and physiological onset mechanisms, as well as the complexity in identifying specific pharmacological targets, make it almost impossible to find effective treatments. Furthermore, because of complex ethical and economic aspects, it is usually hard to find all the necessary resources when searching for drugs for new orphan diseases. In this context, computational methods, based either on receptors or ligands, share the capability to improve the success rate when searching and selecting potential candidates for further experimentation and, consequently, reduce the number of resources and time taken when delivering a new drug to the market. In the present work, a computational strategy based on Molecular Topology, a mathematical paradigm capable of relating the chemical structure of a molecule to a specific biological or pharmacological property by means of numbers, is presented. The result was the creation of a reliable and accessible tool to help during the early in silico stages in the identification and repositioning of potential *hits* for ALS treatment, which can also apply to other orphan diseases. Considering that further computational and experimental results will be required for the final identification of viable *hits*, three linear discriminant equations combined with molecular docking simulations on specific proteins involved in ALS are reported, along with virtual screening of the Drugbank database as a practical example. In this particular case, as reported, a clinical trial has been already started for one of the drugs proposed in the present study.

## 1. Introduction

In the last few decades, basic and clinical research has witnessed a sensible growth in drug design and development using computational methods. Among the main reasons is the necessity for the industry to find more sustainable methods in the process of drug discovery. Computational methods, based on both receptors and ligands, share the capability of improving the success rate when searching and selecting potential candidates for further experimentation, and consequently reduce the number of resources and time spent when delivering a new drug to the market. The importance of such methods is even more relevant in complex scenarios, such as the research in drug design and development for orphan diseases. Due to several reasons which are not a subject of discussion in the present work, such as undefined economic incomes, cryptic information related to the biological and physiological onset mechanisms, or difficulties in identifying specific pharmacological targets, the development of new treatments for orphan diseases is extremely difficult. In the present article, in which, again, it is not the authors’ aim to discuss the ethical aspects and implications of orphan diseases research, the few, actual treatments available for amyotrophic lateral sclerosis treatment (ALS) are collected and a computational strategy based on molecular topology is proposed. 

ALS is a fatal motor neuron disease characterized by degenerative changes in both upper and lower motor neurons [1,2] and its onset typically occurs in late middle life and presents as a relentlessly progressive muscle atrophy and weakness, with the effects on respiratory muscles limiting survival to 2–4 years after disease onset in most cases. ALS prevalence seems to be between 4.1 and 8.4 per 100,000 persons [3]. A slight increase in the prevalence of ALS has been suggested over the years. In the United States, for example, the administrative healthcare data sources and capture–recapture methodology [4] reported an ALS prevalence of 3.7 per 100,000 in 2002, 4.4 per 100,000 in 2003, and 4.8 per 100,000 in 2004. Mehta et al. [5] reported a prevalence of 5.2 per 100,000 in 2015 according to the US National ALS Registry, which was similar to the prevalence of 5.0 per 100,000 as reported in 2014. As mentioned above, the problem with orphan diseases such as ALS lies in the lack of knowledge related to its etiopathology. The specific mechanisms and targets involved in the onset and progression of the disease are undefined; consequently, it is not easy to develop new treatments. To date, some biological targets and gene mutations have been identified in ALS. The TAR DNA-binding protein of 43 KD (TDP-43) [6] and the superoxide dismutase 1 (SOD-1) [7] are two of the most relevant, and many efforts are being made to identify molecules capable of acting on these targets. TDP-43 was identified as a key component of the insoluble and ubiquitinated inclusions in the brains of patients suffering from amyotrophic lateral sclerosis (ALS). The rNLS8 ALS mouse model that exhibits inducible expression of ΔNLS-TDP43 in motor neurons is of particular relevance. This transgenic mouse recapitulates many of the pathologic characteristics of ALS, including cyTDP43 aggregation, motor neuron death, increases in cerebrospinal fluid (CSF) neurofilament levels, neuromuscular junction loss, muscle atrophy, and abnormal compound muscle axon potentials measured by electromyogram [8,9,10]. On the other hand, mutations in the SOD1 gene are responsible for 15% of familial ALS cases and several studies have indicated that SOD1 dysfunction may also play a pathogenic role in sporadic ALS. In terms of treatments available against ALS, over the last decades, more than 40 randomized controlled trials (based in SOD animal models) in patients with ALS failed to show a beneficial effect on disease progression or on survival, illustrating the complexity of the disease [11,12]. All the molecules on the market to date and the recent molecules selected as potential ALS treatments—see, for example, Tofersen [13]—have been tested against SOD-1, showing controversial results. However, the effects of these treatments, as well as the new ones on the new target TAR DNA-binding protein of 43 KD (TDP-43) [14] must be explored. Riluzole and, most recently, Edaravone are the most representative, but neither of them can be considered a real treatment because they are only capable of slightly palliating the symptoms of the disease [15]. 

In this context, QSAR (quantitative structural analysis relationship) and molecular docking were combined to define new theoretical aspects as the basis of an effective strategy for the identification of potential ALS treatments. The trump card of the present computational study was the use of molecular topology, a mathematical paradigm based on graph theory which translates a given chemical structure into a set of mathematical parameters, called topological descriptors [16,17]. The topological descriptors, which are strictly related to the specific molecular connectivity of the molecule under study, are calculated using matrices, flexible and versatile mathematical objects which allow all kinds of operations [18]. Three computational models based on discriminant function analysis (LDA) were developed, along with a molecular docking study performed on two crystalized proteins of TDP-43, retrieved from Protein data bank (PDB) [19,20], to simulate the interaction between the potential candidates identified by molecular topology and the receptor. With the present study, the authors wish to make their contribution to the field of drug design and development for orphan diseases by sharing a reliable and accessible tool to assist during the development of early in silico stages for the identification and repositioning of potential ALS treatment, knowing that further in vitro and in vivo validations will be necessary. 

## 2. Results and Discussion

### 2.1. ALS Models and Internal Validation

In order to identify potential new drugs against ALS, three computational models based on linear discriminant analysis (LDA) have been developed. The first, named the “general model (DF_GEN_)”, focuses on identifying the mathematical pattern to discriminate those ALS treatments which have shown certain activity to those decoys with a degree of structural similarity. The second model, called the “clinical trials model (DF_CLIN_)”, discriminates between treatments with proven activity and those showing lack of activity in ALS clinical trials. The third and final model, named the “promising target TDP-43 model (DF_TDP43_)”, identifies the topological pattern of molecules with activity against the receptor TDP-43. These models consider molecules able to interact with TDP-43 by different mechanisms: reducing stress granule formation such as arcyriaflavin A and ryuvidine, modulation of autophagy such as rapamycin and berberine, targeting the nuclear receptor exportin-1 (XPO1) as KPT-335, reduction of TDP-43 phosphorylation by inhibiting Casein kinase (CK1) such as IGS-2,7 and PHA767491, or by inhibiting Bcr-Abl tyrosine-kinase such as Bosutinib [14].Table 1 shows statistics and descriptors conforming the three developed models. Wider information regarding descriptors value, DF value for training data, and probability of being classified as active by the model is reported in Tables S1–S3 in the Supplementary Material section. Detailed information about internal validation processes can be found in Tables S3 and S4 in the Supplementary Material section.

The classification matrices for all models are presented in Table 2, showing an average correct classification rate above 80% for all models. The DF_GEN_ model reported higher specificity than sensitivity, therefore no false positive was expected. On the contrary, DF_CLIN_ showed a higher sensitivity than specificity, so in this case no false negative was expected. Finally, DF_TDP43_ showed an equal sensitivity and specificity, therefore some false active and negative compounds could be present when applying this model to a virtual screening of databases.

The DF_GEN_ and DF_CLIN_ functions were internally validated using a “leave one out” procedure because the number of compounds belonging to the active groups was low (*n* = 6). As can be seen in Table 2, the results obtained after the internal validation were similar to those presented by the selected models, yielding an average percentage of correct classification for test sets higher than 80% for both models. Therefore, models are robust and their predictions do not depend on the presence of any single compound in the training set.

DF_TDP43_ validation was performed using the “leave some out” method. Data sets were divided into four subgroups: LSO1, LSO2, LSO3, and LSO4, each with approximately 25% of the compounds from both active and inactive/decoys groups. Next, three subgroups were used to build the LDA model, and one of the four subgroups was used as a test set. The process was repeated four times, interchanging the training and prediction subgroups (see Table S4). As can be seen in Table 2, the rate of success in classifying the test set groups was higher than 80%. Therefore, we can again affirm that the model is robust and its predictions do not depend on the presence of a group of compounds that make up the training set data.

In Table 3, all the descriptors used in the construction of the models are listed. As can be seen, there are different types of indices involved, such as 2D autocorrelation, 2D and 3D matrix-based descriptors, connectivity, and geometrical and topological indices.

Next, we will analyze some of the most relevant descriptors when determining the chemical–mathematical pattern related to anti-ALS and anti-TDP-43 activity.

**Figure 1:**
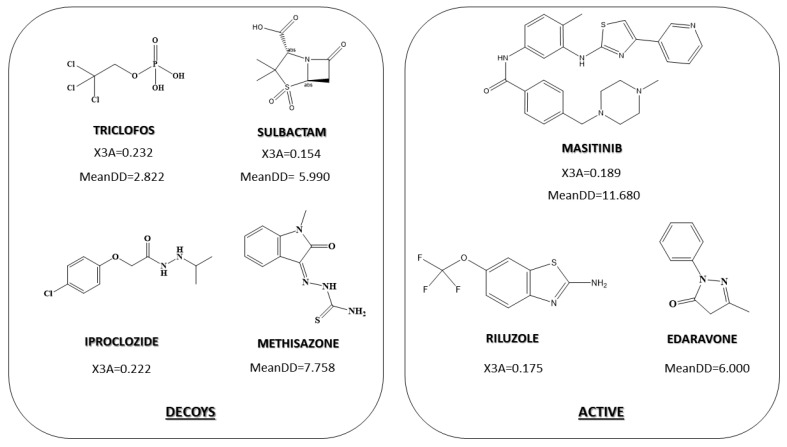
The most relevant descriptors in determining the chemical–mathematical pattern related to anti-ALS activity for DF_gen_.

In the DF_gen_, the X3A and MeanDD indices were those that contribute with a positive sign in the equation, so they were directly related to anti-ALS activity. The higher the value of these descriptors, the greater the probability of presenting anti-ALS activity. The average value of the descriptor X3A in the group of active compounds was 0.181, while for decoys it was 0.189. Although it is not a great difference, we could glimpse how in the group of decoys, in general lines, compounds with an average number of atoms at a distance 3/number of paths at a distance 3 are greater. This translates into a greater presence of less condensed molecules from the topological point of view or, interchangably, more elongated molecules (that is, with a greater presence of linear chains). This can be seen in Figure 1, although it is true that in both groups we found the presence of more condensed and/or elongated molecules.

The other descriptor that contributed positively to the anti-ALS activity of molecules is MeanDD, which considers the length between two atoms of the molecule through the longest path. In this case, the mean value of this descriptor was significantly different between the group of active MeanDD = 8.128 and that of inactive MeanDD = 6.142. This index was generally related to the size of the molecule and its shape (Figure 1). In general, more elongated molecules with a greater presence of cycles adopted a higher value in this descriptor than molecules that are less elongated and formed mainly with aliphatic chains. 

**Figure 2:**
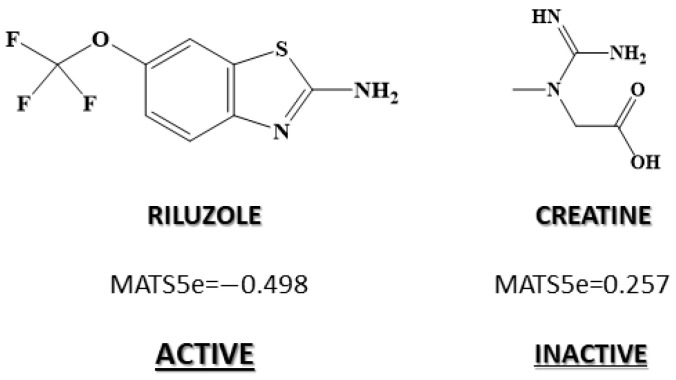
Most relevant descriptors in determining the chemical–mathematical pattern related to anti-ALS tested in clinical trials by DF_clin_.

In the DF_clin_ model, none of the descriptors contributed positively to the equation, so in this case, we analyzed the descriptor that exhibited the highest coefficient (that is, the one that favored non-activity against ALS to a greater degree). MATS5e is a descriptor which considers compounds with electronegative elements at distance 5. Therefore, presence of structural fragments of lag 5, in which the terminal atoms that have high electronegativity would be linked to ALS inactivity. An example is shown in Figure 2, as the inactive creatine label has the highest value for MATS5e index (0.257). This descriptor presented an average value of −0.041 for the inactive group, while the average is −0.264 for the active group, that is to say, clearly lower (Figure 2). Therefore, we can conclude that the group of compounds with demonstrated activity against ALS in clinical trials was made up of molecules with low or no presence of highly electronegative atoms at a distance 5.

**Figure 3:**
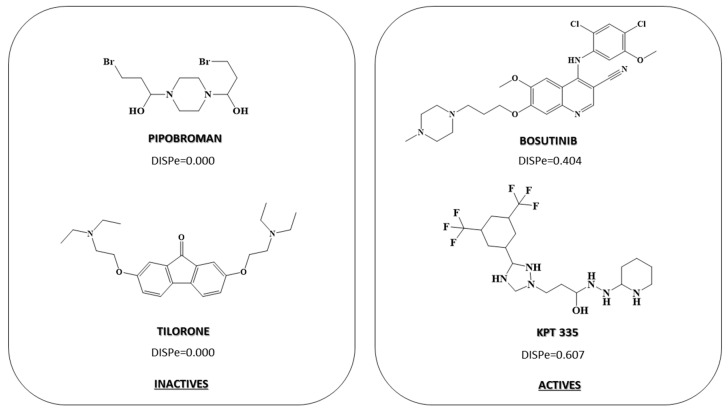
Most relevant descriptors in determining the chemical–mathematical pattern related to anti-TDP-43 activity for DF_TDP43_.

Finally, in the third model, which was focused on the identification of compounds that modulate TDP-43 (DF_TDP43_), we found the DISPe descriptor, which was the one that contributed the most to the activity against TDP-43. This descriptor considers the three-dimensional structure of the molecule, i.e., the position of the atoms in the three-dimensional space. DISPe is defined as the Comparative Molecular Moment Analysis (CoMMA2) value/weighted by atomic Sanderson electronegativities, which represents the displacement between the geometric and the electronegativity centers of the molecule. The positive coefficient of DISPe indicated that molecules with increased displacement between the geometric and the electronegativity centers will show activity against TDP-43. Therefore, as shown in Figure 3, symmetric compounds adopted a zero value for this descriptor while asymmetric compounds with the presence of more electronegative elements (F, Cl, …) adopt higher values of this descriptor, which is why they were associated with active compounds against TDP-43 (Figure 3, compounds Bosutinib and KPT 335). In this descriptor we found a difference between the average value of active drugs compared to TDP-43 DISPe = 0.191 and the inactive ones’ DISPe average = 0.102.

### 2.2. Receiver Operating Characteristic or ROC Curve

**Figure 4:**
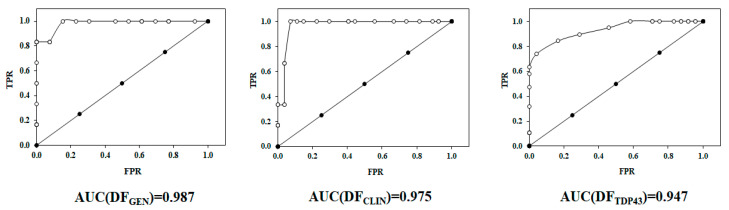
ROC curve for DF_GEN.,_ DF_CLIN_ and DF_TDP43._ TPR: true positive rate; FPR: false positive rate.

In order to assess the reliability of the developed models, the receiver operating characteristic or ROC curve was depicted for each model. The ROC curve provided a graphical plot that illustrates the diagnostic ability of a binary classifier system, as its discrimination threshold is varied. In Figure 4, the ROC curve for all three models is reported. For this discriminant equation, the area under the curve (AUC) value was greater than 0.94 for all models, suggesting a 94% chance that the models correctly distinguished an active and inactive/decoy compound.

### 2.3. Pharmacological Distribution Diagram

**Figure 5:**
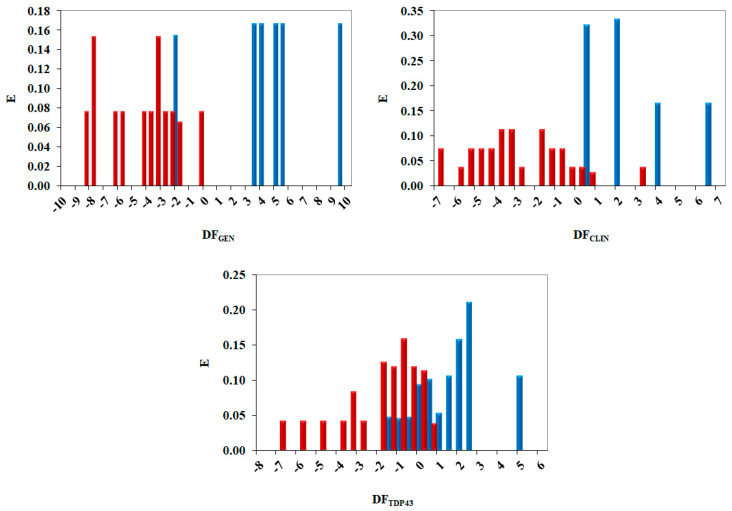
The pharmacological distribution diagram (PDD) for DF_GEN,_ DF_CLIN_ and DF_TDP43_: active compounds are represented by blue bars, whereas inactive/decoys compounds are represented by red bars.

A pharmacological distribution diagram (PDD) provides information about the range of applicability of models. Figure 5 shows the PDD for all models developed, and the distribution of the classification made for training set data (actives and decoys/inactive). 

As can be seen in Figure 5, molecules with reported activity against ALS adopt a DF_GEN_ value above zero, with only one compound below this threshold. Therefore, when this model was applied to the identification of potential ALS drugs, compounds with DF > 0 were selected. Compounds with a DF_GEN_ greater than 10 and lower than −7 were labeled as non-classifiable as they escaped the range of applicability of the model. Additionally, molecules with reported activity in clinical trials showed a DF_CLIN_ value greater than 0.5, even if some inactive compounds exhibited DF values between 0.5 and 1 and 3 and 4. Compounds with a DF_CLIN_ greater than 7 and lower than −10 were labeled as non-classifiable as they escaped the range of applicability of the model. Finally, molecules with activity against the TDP-43 were mainly adopting a DF_TDP43_ value greater than 0; nevertheless, there was an overlapping zone between active and inactive compounds from DF_TDP43_ 0 and 1; therefore, to avoid false active compounds, only compounds with a DF_TDP43_ value greater than 1 were selected as potential TDP-43 compounds. Compounds with a DF_TDP43_ greater than 6 and lower than −8 were labeled as non-classifiable.

### 2.4. Virtual Screening

Once the chemo–mathematical pattern of drugs with reported activity against ALS was determined, it was possible to carry out a virtual screening, searching for novel molecules which might be active against the disease. The molecules needed to fulfill three different requirements (chemo–mathematical pattern): (1) be active against ALS (different mechanisms of action are considered); (2) be potentially active in a clinical trial against ALS, and (3) be capable of interacting with TDP-43 by different mechanisms to reduce TDP-43 activity. In Table S5 of the Supplementary Material, a list of preselected compounds as potential agents against ALS is reported. The Drugbank [21] database was screened and finally, 50 molecules fulfilling at least one chemo–mathematical pattern were selected as potential anti-ALS. From these molecules, a final selection of 10 compounds sharing the same chemo–mathematical pattern as already known ALS active compounds, molecules showing activity in clinical trials for ALS treatment, and molecules interacting with TDP-43 (a promising target on ALS treatment) can be seen further below, in Table 4. From potential anti-ALS compounds selected following the molecular topology strategy, we performed a molecular docking studio to determine its feasibility to link TDP-43 protein and exert the predicted activity as anti-TDP-43 compounds.

### 2.5. Molecular Docking

In order to study how active and decoys/inactive compounds interact with TDP-43 protein, a docking simulation study on already known training set compounds (DF_TDP43_ model) was performed (see Table S6). Only molecules outside overlapping areas of the PDD (Figure 5) were considered; that is, inactive/decoys molecules with DF_TDP43_ between −1.5 and −7 and molecules with reported inhibitory activity against TDP-43 with DF_TDP43_ value between 1 and 5. 

The binding capacity of these molecules was assessed using two different catalytic sites of TDP-43 crystalized protein (PDB: 4IUF [19] and 4BS2 [20]):

- Leu109, Gly110, Pro112, Trp113, and Arg171 (PDB: 4IUF).
- Cys173 and Cys175 (PDB: 4BS2).

**Figure 6:**
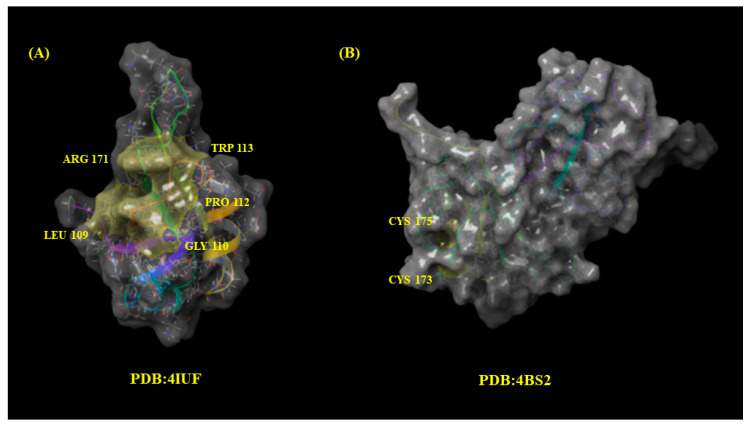
Binding pocket for TDP-43 crystalized protein 4IUF (**A**) and 4BS2 (**B**) with their respective catalytic residues are highlighted in yellow.

As mentioned previously, the altered subcellular localization of trans-active response (TAR) DNA binding protein (TDP-43) and subsequent formation of prion-like TDP-43 aggregated in motor neurons is present in ~95% of patients [22]. TDP-43 mutations have also been identified in patients with familial and sporadic ALS, underscoring the importance of TDP-43 in the pathophysiology of the neurodegeneration seen in this disease [23]. According to literature, 4IUF TDP-43 RNA−Protein Interface has a druggable site in the RRM1 portion [24]. The active site pocket includes residues Leu109, Gly110, Pro112, Trp113, and Arg171 in the RRM1 domain. Among several mutations in the RRM1, mutations Trp113Ala and Arg171Ala seem to be the most deleterious as they increased the estimated dissociation constant of TDP-43. On the other hand, for 4BS2, the oxidative stress related to the cysteninopathia or aberration of cysteine residues modifications seems to be strictly related to the development of ALS. All TDP-43 cysteine residues were suggested as targets: Cys173, Cys175, Cys198, and Cys244 as the major redox-regulated cysteine residues and Cys39 and Cys50 to a much lesser extent. An independent study demonstrated the sequential oxidation of RRM1, with Cys173 being preferentially oxidized and leading to a conformational change allowing Cys175 to be modified and subsequent formation of crosslinked dimers. Analysis of the tandem RRM1-RRM2 structure shows that Cys173 and Cys175 make contacts with residues in the RRM1. Loss of those contacts by oxidation could explain the exposure of amyloidogenic residues 166–173, since Cys173 and 175 were shown to control both correct and aberrant folding of TDP-43 in ALS depending on the freedom of their thiol group [25]. In Figure 6, the 3D surface of the crystalized protein TDP-43 (4IUF and 4BS2) retrieved from PDB is reported. The key catalytic residues of 4IUF and 4BS2 are labeled in yellow.

The results of the docking studio for training set molecules (actives and decoys) are reported in Table S6. When comparing the results for the active compounds during the docking analysis simulation with the results for the decoys (used in the group of inactive), which should be theoretically inactive against TDP-43, only two compounds belonging to the active group did not show interaction with any of the two catalytic sites understudied for crystallographic TDP-43 structure: arcyriaflavin A and panipenam (Table S6). Instead, eight molecules belonging to the decoys did not establish bonds with the reference AA of the two catalytic sites. Therefore, forming interactions with some of the AA of the any catalytic site understudy on 4IUF and 4BS2 will be considered a cut-off point between active and inactive molecules. 

According to the presented chemo–mathematical models, a selection of the most promising potential anti-ALS molecules is reported in Table 4. These molecules satisfied all the three chemical–mathematical functions requirements under study: GEN, CLIN, and TDP43. For the docking simulation, only those molecules classified as active against ALS (DF_GEN_ and DF_CLIN_) and potential inhibitors of TDP-43 are employed.

Potential compounds selected by molecular topology and molecular docking simulation on TDP-43 protein are listed in Table 4, as experimental compounds (i.e., drugs that are at the preclinical or animal testing stage), “investigational” (i.e., drugs that are in human clinical trials), and drugs with different mechanisms of action such as anticancer or antipsychotic agent are selected as promising to treat ALS [26,27,28].

**Table 4:** Potential anti-ALS compounds selected by Molecular Topology and docking score (PDB:4IUF and 4BS2) for TDP-43.

Compounds	DF_GEN_	DF_CLIN_	DF_TDP43_	PDB:4IUF		PDB:4BS2	
				Binding Pocket 1		Binding Pocket 2	
				Docking Score	Amino Acids Interacted	Docking Score	Amino Acids Interacted
9-Methylguanine (DB02489)	11.369	2.217	1.05	−5.076	**Arg171** **(pi-C+ x2)** **Trp113** **(pi-pi)** Leu111 (H) Gly146 (H)	−5.39	**Cys175** **(H x2)** Gln164 (H)
Arimoclomol (DB05025)	3.453	0.364	1.904	−4.761	Lys176 (H, salt) Lys145 (H)	−4.692	Asp174 (2H, salt) Lys176 (H, salt)
Belaperidone	6.004	0.58	0.671	−3.691	**Gly110** **(H)** Asn179 (aroH) Gly146 (H)	−4.014	Asp119 (aroH, H) Asp169 (H) Glu122 (aroH)
Dutasteride	13.278	3.412	0.998	−2.675	Lys145 (H)	−2.843	Asp174 (H)
EGCG (DB12116)	6.849	1.088	4.051	−1.853	Asp174 (H x2) **Arg171** **(pi-C+)** **Gly110** **(H)** Leu111 (H, aroH)	−3.569	**Cys175** **(H)** Asp174 (H, aroH) Arg165 (H)
Levoleucovorin	7.082	3.271	2.234	−3.739	**Trp113** **(aroH)** Gly146 (aroH) Arg165 (halo, H x2) Trp172 (aroH, H) Asp174 (aroH) Leu111 (aro H)	−2.902	Arg165 (H x2) Asp174 (aroH) Lys176 (H, salt)
Neflumozide	8.695	1.587	1.954	−3.598	Ser144 (aroH) **Trp113** **(pi-pi)** Lys145 (H) Gly146 (aroH) **Arg171** (pi-C+)	−4.194	Arg165 (H) Asp174 (salt, H)
Olinciguat (DB15238)	6.699	5.706	1.141	−5.03	**Trp113** **(pi-pi)** Lys145 (aroH) Leu111 (aroH x2) Gly146 (aroH) **Arg171** **(H)** Trp172 (H) Asp174 (H) Arg165 (H x2)	−2.896	**Cys175** **(aroH x2)** Asp174 (aroH) Ser163 (aroH) Arg165 (H x2) Met162 (aroH)
Oxidized coenzyme A (DB01846)	8.410	3.127	1.455	−5.967	Arg165 (H, halo) Trp172 (H) **Arg171** **(H x2, halo, pi-pi)** Lys176 (H,halo x2) Gly146 (H) Asp169 (H)	−5.548	Asp174 (2H) Arg165 (4H,3x salt) Trp172 (H, pi-pi)

EGCG: Epigallocatechin gallate; H: H bond interaction; aroH: aromatic H bond; pi–pi interaction; pi-C+: pi–cation interaction; salt: salt bridge interaction; halo: halogen bond.

**Figure 7:**
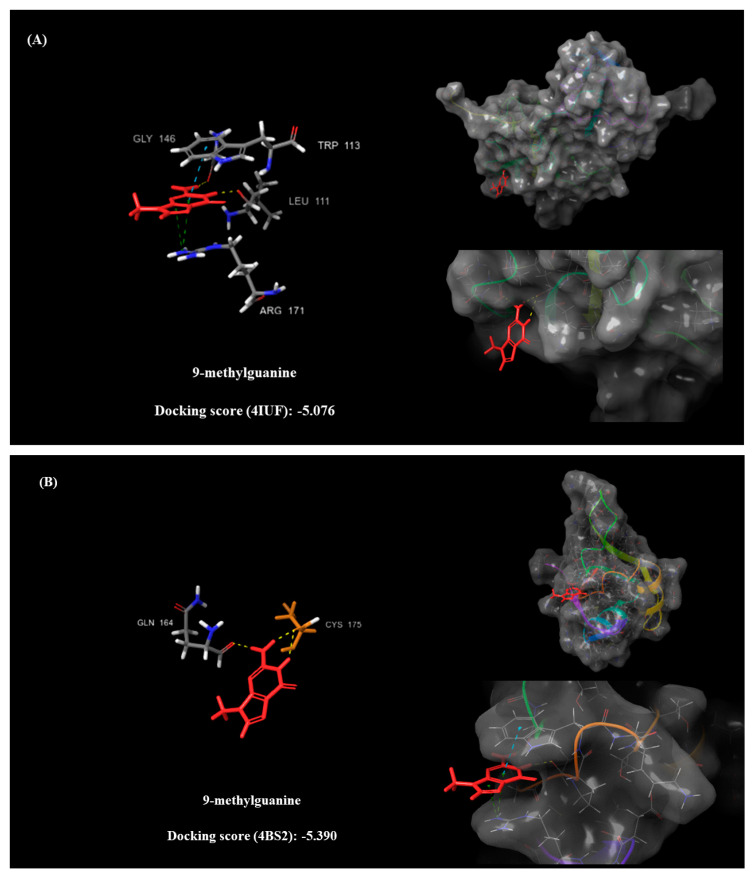
Docking pose and amino acid interaction on 4IUF (**A**) and 4BS2 (**B**) TDP-43 crystalized proteins of 9-methylguanine.

The reported compounds have shown favorable docking scores and stable interactions with relevant amino acids of the TDP-43 crystalized structures (PDB: 4IUF and 4BS2). One of these molecules, arimoclomol, has already been described as a potential ALS treatment in experimental Phase 3 [29] and dutasteride is currently part of an ongoing recent clinical trial, therefore its potential as a treatment for ALS will soon be known [30,31]. Authors cannot exclude other possible activity through the interaction of these molecules with other amino acids of a different active pocket of TDP-43 or dutasteride developing its activity against a completely different target. In Figure 7, the docking pose and amino acid interaction of one of the most promising compounds interacting with the crystallographic structure of TDP-43 is reported (both 4IUF and 4BS2). To date, this xanthine derivative, named 9-methylguanine, is described as an experimental small molecule whose target is dihydroneopterin aldolase, as reported in Drugbank [32] for November 2021. 

According to the present results, 9-methylguanine establishes three hydrogen bonds with key amino acids of the catalytic pocket of TDP-43 crystalized protein (4IUF) and two hydrogen bonds with the Cys175 amino acid residue of the catalytic site of TDP-43 (4BS2), giving insights on its viable profile in targeting a key protein involved in ALS development. Considering all the present computational results, a promising in silico strategy for the identification of potential *hits* against ALS is established; however, in vitro and in vivo experiments will be crucial to corroborate the current results and the reliability of the model. 

## 3. Materials and Methods

### 3.1. Analysis of Dataset Compounds and Search Algorithm

The dataset used in the construction of the three discriminant models was collected from literature [14,33,34,35]. Molecules were retrieved from online chemical databases, such as Chemspider [36] or designed *ad hoc*, using ChemDraw software from PerkinElmer [37]. A total of 93 active, inactive or decoy molecules were collected. Structural similarity between active and inactive/decoys sets was determined by the Tanimoto coefficient and visual inspection in order to assess a reliable discrimination model (DF_GEN_ and DF_TDP43_). The set of inactive/decoys compounds were selected from CMC databases [38]. All the data are reported in the Supplementary Material section. 

**Figure 8:**
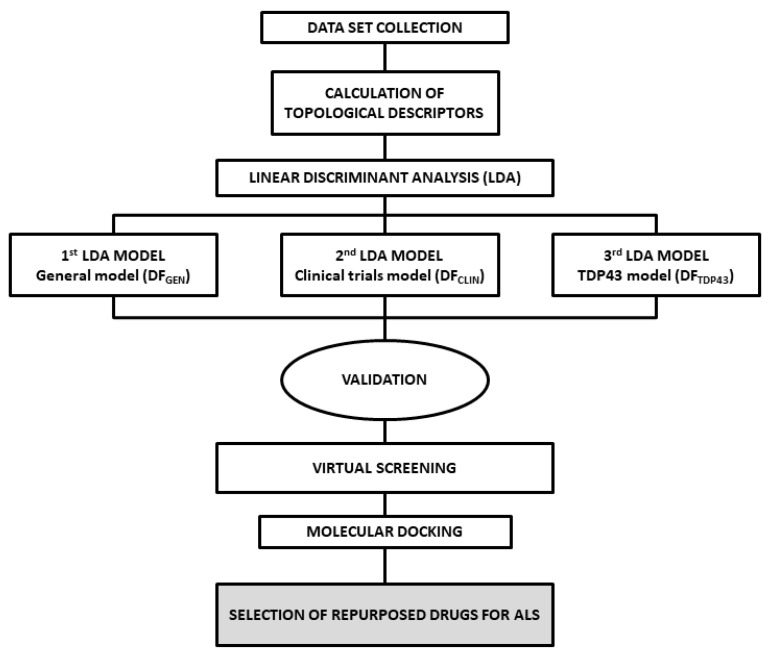
Search algorithm used to develop the in silico strategy for the repositioning of potential ALS drugs.

In Figure 8, the search algorithm strategy developed in the present study for the identification of potential ALS treatments is reported.

The topological and topochemical indices were calculated using alvaDesc software [39] (version 2) and all their values for the selected equations for each compound included in the study (training set) are shown in the Supplementary Material section. 

### 3.2. Statistical Modeling Methods

Statistical methods are essential when searching for strong and reliable predictive models. The linear algorithms used in predictive equations, such as Linear Discriminant Analysis (LDA), allow a linear combination of features to separate two or more classes of objects in specific classification categories. In the present study, the LDA is employed to generate three different reliable discriminant models, which, when combined, should provide enough information to repurpose drugs with potential anti-ALS activity: (1) general model (DF_GEN_); (2) clinical trials model (DF_CLIN_) and (3) TDP43 model (DF_TDP43_). The most important aspect of a robust LDA model is the selection of the most significant variables or descriptors which will characterize the compounds and their ALS activity or inactivity. Selection of the best descriptors is made here by following the forward stepwise algorithm based on *p*-value, therefore in each step the variable with a more favorable *p*-value < 0.05 is selected and so on. The process finishes when the algorithm cannot introduce anymore descriptors with a *p*-value less than 0.05. Therefore, at each step, the variable that adds the most to the separation of the groups is entered into the discriminant function. The significance of the selected descriptor could be addressed by the Fisher–Snedecor parameter [40], the more significance, the higher value adopts. The quality of the discriminant function is assessed by the Wilks’ lambda parameter [40]. Generally speaking, the Wilks’ lambda can take values between 0 and 1, and the smaller the value, the better the prediction. Statistica was the software used for developing linear discriminant models [41].

### 3.3. ALS Models and Validation

#### 3.3.1. Classification Matrix, LOO and LSO Validation

The discriminant reliability of the LDA models was evaluated following two methods. The first was the classification matrix, which sorts all cases from the model into categories by determining whether the predicted value matches the actual value. The number of cases classified into each group and the percentage of correct classifications are reported in the Supplementary Material. The predictive power of the model was checked using the “leave one out” (LOO) jackknife cross-validation strategy [42,43]. In the LOO algorithm, one compound is eliminated from the dataset and the discriminant analysis, with the N-1 remaining compounds and the original descriptors, is performed again. Over the calculation of the remaining compounds, the previously removed case is then classified. “Leave some out” (LSO) cross-validation follows the same procedure as LOO, but instead of just leaving one compound out, it involves leaving a percentage of the training set out. 

#### 3.3.2. ROC Curve

To assess the predictive capability of the LDA models and determine their sensitivity and specificity, the relative operating characteristic curve or ROC curve was calculated [44]. The sensitivity is intended as the true positive rate and is defined as the percentage of active molecules correctly classified by the model, while specificity, or true negative rate, is the percentage of no-active molecules correctly classified by the model. In the ROC curve the *y*-axis represents the sensitivity of the model as the discrimination threshold is varied, which simultaneously affects the specificity of the model. For convenience, the *x*-axis represents 1-specificity, so that both magnitudes change in the same direction as the discrimination threshold is varied. In this context, the area under the ROC curve (AUC) is often regarded as an indicator of the performance of the classifier. A value of AUC = 1 would be obtained for a perfect classifier, whereas the diagonal line would represent a model with no classification power in predicting binary outcomes. The best possible prediction method would yield a point in the upper left corner or coordinate (0,1) of the ROC space, representing 100% sensitivity (no false negatives) and 100% specificity (no false positives). The (0,1) point is also called a perfect classification. A random guess would give a point along a diagonal line (the so-called line of no discrimination) from the left bottom to the top right corner (regardless of the positive and negative base rates).

### 3.4. Pharmacological Distribution Diagram

Linear discriminant analysis in topological QSAR enables the plotting of frequency distribution diagrams, called pharmaceutical distribution diagrams [45]. The diagrams represent a frequency of the number of molecules within an interval of values of the discriminant function vs. these values. The plot provides a straightforward way of visualizing the regions of minimum overlap between active and inactive compounds, DF regions, with the highest expectancy of finding active molecules and range of applicability domain for a DF. For an arbitrary range of values of a given function, an “expectancy of activity” can be defined as E_a_ = a/(i + 1), where “a” is the number of active compounds in the range divided by the total number of active compounds and “i” is the number of inactive compounds in the interval divided by the total number of inactive compounds. The expectancy of inactivity is defined in a symmetrical way, as E_i_ = i/(a + 1). 

### 3.5. Virtual Screening

Virtual screening of the Drugbank database [21] was used to identify potential repurposed drugs against ALS. A stepwise strategy was adopted, performing a virtual screening using each one of the models described (DF_GEN_, DF_CLIN_ and DF_TDP43_). Finally, a docking studio was performed on potential anti-ALS repurposed drugs in order to address its mechanism of action. 

### 3.6. Molecular Docking

The crystal structure of TDP-43 RRM1-DNA complex, also called 4IUF [19], was retrieved from the Protein Data Bank (PDB) along with the 4BS2 UG-rich RNA TDP-43 [20]. To perform the molecular docking simulations, Maestro software from Schrödinger Suite was employed [46]. The key residues of the catalytic site were identified and the protein was prepared. Although water can play a crucial role in docking simulations, especially water next to the interaction pocket, in the present study, the protein was prepared by removing water in order to avoid unwanted interactions. Of course, this aspect must be considered when evaluating the simulation results. Docking was performed on the active region by assigning specific grid-box coordinates. For each molecule, the “five best subset binding scores” were calculated, reporting the free Gibbs energy value and the number of HBs (hydrogen bonds) between the molecule and the residues of the catalytic site along with the bond length. 

## 4. Conclusions

In the present study, pattern recognition analysis was developed to identify a specific chemo–mathematical pattern for ALS treatments, using the few drugs that have thus far shown activity against different therapeutic targets. The most promising target seems to be TDP-43, whose modulation seems to alleviate ALS symptoms and progression. After the construction of three predictive QSAR models based on linear discriminant analysis for the identification of molecules with potential activity in ALS and targeting TDP-43, the models were validated and applied to the virtual screening of the Drugbank database with the aim of redirecting known drugs to ALS. Ten compounds were finally identified, of which one was already known and the other is currently part of an ongoing clinical trial for ALS. As for the other eight compounds, in vitro and in vivo tests will be crucial to determine their activity as anti-ALS and to corroborate the reliability of the in silico strategy. As far as the authors are aware, this is the first work in which molecular topology is used to identify a common chemo–mathematical pattern for ALS treatments and to redirect known drugs for the treatment of symptoms linked to ALS.

## Figures and Tables

**Table 1:** Equations of the different discriminant models developed, statistical parameters and internal validation data.

Model Data	Internal Validation
$DF_{GEN}=\left(1.916\times MeanDD\right)-\left(21.611\times MATS5m\right)+\left(151.793\times X3A\right)-\left(695.461\times VE2sign\_\_\;D\right)-39.907$ N = 19, λ = 0.321, F = 7.388, *p* < 0.002	LOOval:
	λ_CV_ = 0.318
	F_CV_ = 7.093
	*p*_cv_ < 0.004
$DF_{CLIN}=\left(-5.443\times SM1\_DZ\left(p\right)\right)-\left(0.189\times ATSC8m\right)-\left(0.222\times ATSC3m\right)-\left(11.512\times MATS5e\right)-12.587$ N = 33, λ = 0.525, F = 6.339, *p* < 0.0009	LOO val:
	λ_CV_ = 0.522
	F_CV_ = 6.217
	p_cv_ < 0.001
$DF_{TDP43}=\left(-6.67\times VE1sign_{Dz\left(p\right)}\right)+\left(12.37\times DISPe\right)-\left(1.08\times J\_G\right)+3.59$ N = 43, λ = 0.530, F = 11.510, *p* < 0.00001	LSO val:
	λ_CV_ = 0.509
	F_CV_ = 9.217
	*p*_cv_ < 0.0003

N: number of molecules; λ: Wilks’ lambda; F: Fischer–Snedecor parameter; *p*: *p*-value or probability value; LOO: leave one out; LSO: leave some out; val: validation; cv: cross-validation; MeanDD: mean pairwise detour distance; MATS5m: Moran autocorrelation of lag 5 weighted by mass; X3A: average connectivity index of order 3; VE2sign_D: average coefficient of the last eigenvector from topological distance matrix; SM1_DZ(p): spectral moment of order 1 from Barysz matrix weighted by polarizability; ATSC8m: centred Broto–Moreau autocorrelation of lag 8 weighted by mass; ATSC3m: centred Broto–Moreau autocorrelation of lag 3 weighted by mass; MATS5e: Moran autocorrelation of lag 5 weighted by Sanderson electronegativity; VE1signDz(p): sum of the last eigenvector from Barysz matrix weighted by polarizability; DISPe: displacement value/weighted by Sanderson electronegativity; J_G: Balaban-like index from geometrical matrix.

**Table 2:** Classification matrices for the ALS models and internal validation (LOO and LSO).

	Model				Internal Validation
		% of Correct Classification	Active	Inactive	% of Correct Classification
	Active	83.3	5	1	95.9 ^LOO^
**DF_GEN_**	Inactive	100.0	0	13	
	Average	91.7			
	Active	100.0	6	0	84.3 ^LOO^
**DF_CLIN_**	Inactive	88.9	3	24	
	Average	94.5			
	Active	84.2	16	3	81.7 ^LSO^
**DF_TDP43_**	Inactive	83.3	4	20	
	Average	83.8			

**Table 3:** Topochemical descriptors used in the construction of ALS models are presented below.

Descriptor Type	Descriptor Name	Descriptor Definition
2D autocorrelations index	MATS5m	Moran autocorrelation of lag 5 weighted by mass
2D autocorrelations index	MATS5e	Moran autocorrelation of lag 5 weighted by Sanderson electronegativity
2D autocorrelations index	ATSC3m	Centered Broto–Moreau autocorrelation of lag 3 weighted by mass
2D autocorrelations index	ATSC8m	Centered Broto–Moreau autocorrelation of lag 8 weighted by mass
2D matrix-based descriptors	VE1sign_Dz(p)	Sum of the last eigenvector from Barysz matrix weighted by polarizability
2D matrix-based descriptors	VE2sign_D	Average coefficient of the last eigenvector from topological distance matrix
2D matrix-based descriptors	SM1_DZ(p)	Spectral moment of order 1 from Barysz matrix weighted by polarizability
3D matrix-based descriptors	J_G	Balaban-like index from geometrical matrix
Connectivity index	X3A	Average connectivity index of order 3
Geometrical descriptors	DISPe	Displacement value/weighted by Sanderson electronegativity
Topological index	MeanDD	Mean pairwise detour distance

## Data Availability

Data is contained within the article or supplementary material.

## Supplementary Materials

The following supporting information can be downloaded at: https://www.mdpi.com/article/10.3390/ph15010094/s1. For the three discriminant models, descriptors and DF value, probability of being classified as active by the model before and after LOO internal validation procedure for all the training set molecules are reported in Tables S1–S3. LSO internal validation procedure for DF_TDP43_ is reported in Table S4. Results of the Drugbank database virtual screening are reported in Table S5. In Table S6, TDP43 inhibitors and decoys from DF_TDP43_ training set employed as references for molecular docking analysis are detailed.
