# Supplementary material for: How Molecular Topology Can Help in Amyotrophic Lateral Sclerosis (ALS) Drug Development: A Revolutionary Paradigm for a Merciless Disease
Source: Pharmaceuticals (Basel). 2022 Jan 14;15(1):94. doi: 10.3390/ph15010094 (PMC8781553; doi:10.3390/ph15010094)
Supplement: Supplementary file 1 [file pharmaceuticals-15-00094-s001.zip › pharmaceuticals-1478736-supplementary.pdf]

Molecular topology may bring some hope in amyotrophic lateral sclerosis (ALS) treatment development. A revolutionary paradigm for a ruthless disease

Maria Galvez-Llompart[1], Riccardo Zanni[2], Ramon Garcia-Domenech[2], Jorge Galvez[2*]

[1]*Instituto de Tecnología Química, UPV-CSIC, Universidad Politécnica de Valencia, Valencia, Spain. E-mail:* *magllo@itq.upv.es*

[2*]*Molecular Topology and Drug Design Unit, Department of Physical Chemistry, University of Valencia, Valencia, Spain. E-mail:* *jorge.galvez@uv.es*

Supplementary Material

Table S1. Descriptors and DF_{GEN} value, probability of being classified as active by the model and after LOO internal validation procedure.

Compound	MeanDD	X3A	VE2sign_D	MATS5m	Prob(activ)	DF_{GEN}	$Prob(activ)_{LOO}$
				**Active group**			
**Edaravone**	6.000	0.185	0.005	-0.405	0.994	5.075	0.972
**Kopal**	9.882	0.177	0.001	-0.215	1.000	9.590	1.000
**Masitinib**	11.680	0.189	0.003	0.170	0.996	5.608	0.936
**Riluzole**	7.229	0.175	0.006	-0.369	0.985	4.161	0.967
**Telbivudine**	6.441	0.185	0.003	0.027	0.135	-1.854	0.967
**Tirasemtiv**	7.537	0.176	0.001	-0.155	0.975	3.656	0.961
				**Inactive group**			
**Cilutazoline**	7.022	0.183	0.010	-0.113	0.039	-3.211	0.057
**Cotinine**	6.013	0.191	0.005	0.127	0.003	-5.753	0.004
**Fludorex**	5.392	0.192	0.006	-0.034	0.024	-3.698	0.032
**Fluminorex**	6.100	0.186	0.001	0.102	0.070	-2.579	0.027
**Gacyclidine**	6.902	0.168	0.005	-0.224	0.467	-0.131	0.651
**Iproclozide**	5.767	0.222	0.005	0.204	0.034	-3.341	0.108
**Methisazone**	7.758	0.178	0.017	-0.106	0.001	-7.532	0.000
**Panidazole**	6.853	0.191	0.009	0.127	0.002	-6.490	0.002
**Pemoline**	6.000	0.185	0.005	-0.093	0.156	-1.683	0.182
**Rufinamide**	6.779	0.191	0.006	0.102	0.018	-4.024	0.023
**Sulbactam**	5.990	0.154	0.009	-0.148	0.000	-7.956	0.000
**Triclofos**	2.822	0.232	0.000	0.414	0.000	-8.203	0.000
**Uridine**	6.449	0.183	0.002	0.032	0.100	-2.195	0.140

red colour: misclassified compounds by the model.

Table S2. Descriptors and DF_{CLIN} value, probability of being classified as active by the model and after LOO internal validation procedure.

Compound	SM1_Dz(p)	ATSC3m	ATSC8m	MATS5e	P.A.	DF_{CLIN}	$P.A._{LOO}$
**Active group**							
Edaravone	-1.224	10.600	3.198	-0.437	0.700	0.848	0.416
Kopal	-2.568	16.180	22.678	-0.303	0.985	4.170	0.978
Masitinib	-2.153	36.928	27.510	-0.015	0.908	2.286	0.719
Riluzole	-2.244	6.777	0.393	-0.498	0.999	6.786	0.999
Telbivudine	-2.104	19.021	9.363	-0.087	0.910	2.317	0.869
Tirasemtiv	-1.526	15.494	6.382	-0.243	0.678	0.747	0.623
**Inactive group**							
Bromocriptine	-2.259	61.361	81.203	-0.042	0.175	-1.553	0.205
Ceftriaxone	-2.567	18.986	36.936	-0.109	0.466	-0.137	0.614
Celecoxib	-2.419	15.813	21.162	-0.023	0.587	0.353	0.671
Coenzyme_Q10	-1.758	76.808	97.011	-0.004	0.014	-4.286	0.015
Creatine	-1.649	7.914	0.000	0.257	0.008	-4.814	0.008
Dextromethorphan	-1.030	30.612	14.058	0.053	0.030	-3.467	0.036
Diazoxide	-1.390	10.048	5.076	-0.044	0.038	-3.242	0.042
Erythropoietin	-1.536	24.388	31.665	-0.045	0.014	-4.282	0.015
Gacyclidine	-0.188	31.842	8.624	-0.192	0.019	-3.924	0.022
Guanabenz	-1.103	9.340	15.024	-0.052	0.001	-6.756	0.001
IGF-1	-3.515	151.457	233.752	0.104	0.005	-5.276	0.000
Lamotrigine	-1.285	10.082	7.267	-0.163	0.055	-2.853	0.063
Malondialdehyde	-1.224	3.741	0.000	0.000	0.006	-5.097	0.006
Memantine	-0.470	26.516	0.000	-0.640	0.962	3.222	1.000
Minocycline	-2.416	32.944	40.940	-0.041	0.644	0.592	0.706
Nimesulide	-2.055	14.355	16.269	0.198	0.027	-3.569	0.032
N-methyl-D-aspartate	-1.856	10.003	0.000	0.249	0.042	-3.131	0.053
Olesoxime	-1.030	51.467	43.298	0.014	0.019	-3.925	0.022
Pioglitazone	-1.688	26.799	16.603	0.015	0.316	-0.772	0.370
Pramipexole	-0.878	22.226	8.248	0.038	0.008	-4.879	0.008
Pyrimethamine	-1.165	15.400	12.599	-0.343	0.220	-1.265	0.282
Resveratrol	-1.526	12.386	8.006	-0.137	0.187	-1.473	0.199
Talampanel	-1.856	21.748	26.354	-0.076	0.145	-1.772	0.156
TCH346	-1.030	17.725	15.741	-0.026	0.003	-5.728	0.003
Valproic_acid	-1.224	13.105	1.285	-0.153	0.182	-1.502	0.195
Vitamin_D	-0.788	45.597	45.194	0.019	0.001	-6.949	0.001
Xaliproden	-2.083	25.502	25.363	0.029	0.325	-0.729	0.360

red colour: misclassified compounds by the model.

Table S3. Descriptors and DF_{TDP43}'s value, probability of being classified as active by the model.

Compound	VE1sign_Dz(p)	DISPe	J_G	DF_{TDP43}	Class.	P.A.
**Active**						
Arcyriaflavin A	0	0.115	2.217	2.61	A	0.932
Berberine	0.098	0.119	1.678	2.59	A	0.93
Bosutinib	0.665	0.404	1.963	2.031	A	0.884
CHC	0.082	0.17	2.479	2.467	A	0.922
Furomazine	0.338	0.082	3.318	-1.23	I	0.226
Ibacitabine	0.281	0.038	2.654	-0.684	I	0.335
Idoxuridine	0.276	0.128	3.001	0.088	A	0.522
IGS-2,7	0.082	0.17	2.479	2.467	A	0.922
KPT 335	0.419	0.607	2.961	5.096	A	0.994
LND-0130436	0.152	0.154	4.293	-0.157	I	0.461
Olomoucine	0.114	0.176	2.144	2.686	A	0.936
Panipenam	0.122	0.193	3.45	1.439	A	0.808
PHA767491	0.046	0.069	3.027	0.864	A	0.704
Pioglitazone	0.178	0.289	3.109	2.617	A	0.932
SB 415286	0.193	0.13	3.436	0.189	A	0.547
SB216763	0.009	0.316	2.013	5.261	A	0.995
3-((6,7-dimethoxyquinazolin-4-yl)amino)phenol	0.048	0.062	3.237	0.539	A	0.632
Tropapride	0.006	0.117	2.946	1.811	A	0.86
Vorinostat	0.243	0.298	3.535	1.831	A	0.862
**Inactive**						
4-carbamoyl-2'-hydroxyiminomethyl-1,1'-oxidimethylenedi(pyridinium) dichloride	0.027	0.086	4.125	0.019	A	0.505
Amphotericin B	0.028	0.125	10.728	-6.644	I	0.001
Clometacin	0.335	0.09	3.344	-1.143	I	0.242
Cloxypendyl	0.645	0.081	3.24	-3.207	I	0.039
Fipronil	0.168	0.096	3.982	-0.646	I	0.344
Flumedroxone-17-acetate	0.286	0.187	4.176	-0.519	I	0.373

Fluperamide	0.313	0.33	4.471	0.753	A	0.680
Maduramicin	0.025	0.077	5.099	-1.138	I	0.243
Metibride	0.34	0.145	3.01	-0.14	I	0.465
Mizolastine	0.354	0.028	2.978	-1.649	I	0.161
Nelzarabine	0.281	0.076	2.174	0.299	A	0.574
Oxazafone	0.637	0.066	4.545	-4.748	I	0.009
Parconazole	0.674	0.037	3.06	-3.758	I	0.023
Perfomedil	0.219	0.16	4.862	-1.144	I	0.242
Pipobroman	0	0	4.85	-1.647	I	0.162
Pirifibrate	0.025	0.032	3.532	0.003	A	0.501
Pivoxazepam	0.626	0.096	3.196	-2.847	I	0.055
Sertindole	0.611	0.05	3.347	-3.489	I	0.03
Temelastine	0.101	0.046	3.789	-0.607	I	0.353
Thioinosine	0.32	0.063	2.33	-0.288	I	0.429
Tiaramide	0.438	0.105	3.453	-1.761	I	0.147
Tilorone	0	0.064	4.939	-0.953	I	0.278
Uldazepam	0.99	0.054	2.938	-5.516	I	0.004
Vinconate	0.568	0.178	2.146	-0.324	I	0.42

Table S4. LSO internal validation procedure for DF_{TDP43} (1=training active group; 2=training inactive group; 3=test active group; 4=test inactive group).

Compound	Class. DF_{TDP43}	P.A.	Class. LSO1	P.A. LSO1	Class. LSO2	P.A. LSO2	Class. LSO3	P.A. LSO3	Class. LSO4	P.A. LSO4
Arcyriaflavin A	1	0.932	1	0.954	1	0.928	1	0.989	3	0.884
Berberine	1	0.930	3	0.952	1	0.921	1	0.993	1	0.877
Bosutinib	1	0.884	1	0.768	3	0.919	1	0.968	1	0.850
CHC	1	0.922	1	0.935	1	0.927	3	0.980	1	0.879
Furomazine	1	0.226	1	0.322	3	0.141	1	0.200	1	0.221
Ibacitabine	1	0.335	1	0.497	1	0.203	3	0.516	1	0.292
Idoxuridine	1	0.522	1	0.600	1	0.442	1	0.619	3	0.476
IGS-2,7	1	0.922	1	0.935	1	0.927	1	0.980	3	0.879
KPT 335	1	0.994	3	0.971	1	0.999	1	0.997	1	0.991
LND-0130436	1	0.461	1	0.520	1	0.435	1	0.232	3	0.461
Olomoucine	1	0.936	1	0.946	1	0.940	3	0.989	1	0.895
Panipenam	1	0.808	1	0.823	3	0.824	1	0.829	1	0.767
PHA767491	1	0.704	1	0.811	1	0.635	1	0.824	3	0.632
Pioglitazone	1	0.932	1	0.911	1	0.955	3	0.961	1	0.907
SB 415286	1	0.547	1	0.627	3	0.489	1	0.541	1	0.510
SB216763	1	0.995	1	0.993	1	0.998	3	0.999	1	0.989
3-((6,7-dimethoxyquinazolin-4-yl)amino)phenol	1	0.632	1	0.762	3	0.548	1	0.718	1	0.567
Tropapride	1	0.860	3	0.903	1	0.851	1	0.938	1	0.803
Vorinostat	1	0.862	3	0.817	1	0.902	1	0.854	1	0.836
4-carbamoyl-2'-hydroxyiminomethyl-1,1'-oxidimethylenedi(pyridinium) dichloride	2	0.505	4	0.634	2	0.444	2	0.339	2	0.481
Amphotericin b	2	0.001	2	0.002	2	0.001	4	0.000	2	0.005
Clometacin	2	0.242	2	0.334	2	0.156	2	0.211	4	0.236
Cloxypendyl	2	0.039	2	0.058	4	0.016	2	0.023	2	0.047
Fipronil	2	0.344	2	0.455	4	0.269	2	0.203	2	0.339
Flumedroxone-17-acetate	2	0.373	2	0.393	2	0.346	4	0.169	2	0.386
Fluperamide	2	0.680	2	0.568	4	0.765	2	0.355	2	0.691
Maduramicin	2	0.243	2	0.360	2	0.192	2	0.044	4	0.269
Metibride	2	0.465	2	0.525	2	0.387	4	0.440	2	0.430
Mizolastine	2	0.161	4	0.278	2	0.079	2	0.190	2	0.153
Nelzarabine	2	0.574	2	0.695	2	0.445	4	0.846	2	0.490
Oxazafone	2	0.009	2	0.014	2	0.003	4	0.001	2	0.014
Parconazole	2	0.023	2	0.041	2	0.008	2	0.015	4	0.028
Perfomedil	2	0.242	2	0.280	2	0.213	2	0.047	4	0.276
Pipobroman	2	0.162	2	0.314	2	0.098	4	0.035	2	0.177
Pirifibrate	2	0.501	4	0.678	2	0.391	2	0.507	2	0.452
Pivoxazepam	2	0.055	4	0.078	2	0.025	2	0.036	2	0.064

Sertindole	2	0.030	2	0.051	2	0.011	2	0.015	4	0.036
Temelastine	2	0.353	4	0.416	2	0.253	2	0.264	2	0.333
Thioinosine	2	0.429	4	0.470	2	0.288	2	0.698	2	0.366
Tiaramide	2	0.147	2	0.200	4	0.087	2	0.097	2	0.155
Tilorone	2	0.278	2	0.417	2	0.219	2	0.065	4	0.296
Uldazepam	2	0.004	2	0.006	4	0.001	2	0.002	2	0.006
Vinconate	2	0.420	2	0.437	4	0.323	2	0.680	2	0.376

red colour: misclassified compounds by the model.

Table S5. Virtual screening of Drugbank database: selection of potential anti-ALS determined by MT strategy. Highlight in grey compounds fulfilling all three models.

Compound	DF_{GEN}	Class	P.A.	DF_{CLIN}	Class	P.A.	DF_{TDP43}	Class	P.A.
9Methylguanine	11.369	A	1.000	2.217	A	0.902	1.05	A	0.870
Alpiropride	-3.082	I	0.044	1.336	A	0.792	-1.914	I	0.129
Amosulalol	4.713	A	0.991	2.344	A	0.913	-2.977	I	0.049
Arimoclomol	3.453	A	0.969	0.364	A	0.590	1.904	A	0.870
Arzoxifene	2.319	A	0.910	-1.503	I	0.182	-3.040	I	0.046
Atrimustine	18.545	A	1.000	3.675	A	0.975	-2.213	I	0.099
Balaperidone	6.004	A	0.998	0.580	A	0.641	0.671	A	0.662
Couroupitine A	15.179	A	1.000	-0.852	I	0.299	3.126	A	0.958
Doconazole	0.498	A	0.621	-1.916	I	0.128	1.218	A	0.772
Dotarizine	5.400	A	0.995	-2.465	I	0.078	0.021	I	0.505
Dutasteride	13.278	A	1.000	3.412	A	0.968	0.998	A	0.731
EGCG	6.849	A	0.999	1.088	A	0.748	4.051	A	0.983
Etamestrol	13.257	A	1.000	0.390	A	0.596	-2.446	I	0.080
Etoprine	2.597	A	0.931	-1.871	I	0.133	0.544	A	0.633
Fenfluthrin	-0.600	I	0.354	2.762	A	0.941	2.542	A	0.927
Formycin	1.268	A	0.780	1.381	A	0.799	-0.920	I	0.285
Fuprazole	6.527	A	0.999	-1.939	I	0.126	-1.074	I	0.255
Halometasone	12.007	A	1.000	3.469	A	0.970	0.093	I	0.523
Icospiramide	2.811	A	0.943	0.531	A	0.630	-0.260	I	0.436
Idoxuridine	-1.957	I	0.124	-0.674	I	0.338	0.615	A	0.649
Imiclopazine	5.731	A	0.997	-2.919	I	0.051	-2.689	I	0.064
Ipsapirone	7.104	A	0.999	3.273	A	0.964	-0.347	I	0.414
Ketoconazole	0.860	A	0.702	0.600	A	0.646	-1.521	I	0.179
Lamotrigine	3.384	A	0.967	-2.853	I	0.055	1.952	A	0.876
Levoleucovorin	7.082	A	0.999	3.271	A	0.963	2.234	A	0.903
Mazipredone	8.855	A	1.000	0.972	A	0.726	-3.642	I	0.026
Metopimazine	1.876	A	0.867	-2.181	I	0.102	-3.883	I	0.020
Metoprine	3.406	A	0.968	-0.776	I	0.315	1.396	A	0.802
Mycanodin	-5.291	I	0.005	-8.099	I	0.000	1.119	A	0.754
Naftopidil	12.392	A	1.000	1.011	A	0.733	0.479	I	0.618
Neflumozide	8.695	A	1.000	1.587	A	0.830	1.954	A	0.876
Nicogrelate	2.205	A	0.901	-1.417	I	0.195	0.989	A	0.729
Nitramisole	-2.501	I	0.076	-1.811	I	0.140	4.353	A	0.987
Ocaperidone	11.026	A	1.000	-0.116	I	0.471	1.957	A	0.8761
Olinciguat	6.699	A	0.999	5.706	A	0.997	1.141	A	0.758
Oxaflumazine	5.551	A	0.996	1.595	A	0.832	-1.515	I	0.180
Oxidized coenzyme A	8.410	A	1.000	3.127	A	0.958	1.455	A	0.811
Panipenam	-4.873	I	0.008	2.223	A	0.902	1.439	A	0.808
Piretanide	-5.988	I	0.002	-2.821	I	0.056	2.389	A	0.916
Pyrazofurin	-2.063	I	0.113	2.707	A	0.937	0.243	I	0.561
Revospirone	6.029	A	0.998	2.558	A	0.928	-0.663	I	0.340

Rofelodine	-0.550	I	0.365	-2.841	I	0.055	1.958	A	0.876
Rosiglitazone	4.135	A	0.984	-0.257	I	0.436	0.670	A	0.662
Sabeluzole	5.890	A	0.997	-0.739	I	0.323	-0.695	I	0.333
Spiropiperidine	9.676	A	1.000	-1.428	I	0.193	-0.245	I	0.439
Tamsulosin	7.407	A	0.999	1.032	A	0.737	-2.836	I	0.055
Tefludazine	-1.267	I	0.219	0.889	A	0.709	-2.241	I	0.096
Terconazole	-0.414	I	0.397	0.158	A	0.540	-0.347	I	0.414
Traxanox	5.405	A	0.996	-1.883	I	0.132	3.325	A	0.965
Valperinol	-7.398	I	0.001	0.791	A	0.688	-0.491	I	0.380

EGCG: Epigallocatechin gallate

Table S6. TDP43 inhibitors and decoys from DF_{TDP43} training set employed as reference for docking studio.

**Compounds**	**PDB:4IUF**		**PDB:4BS2**	
	**Binding pocket 1**		**Binding pocket 2**	
	**Docking score**	**Amino acids interacted**	**Docking score**	**Amino acids interacted**
	**Active**			
Arcyriaflavin A	-3.472	Cys145<br>(pi-pi)<br>Asp138<br>(H)	-3.425	Asp174<br>(H)
Berberine	**-3.999**	**Trp113**<br>**(pi-pi)**<br>Phe147<br>(pi-pi)<br>Gly146<br>(aroH x2)<br>**Arg171**<br>**(pi-cation)**<br>Leu111<br>(aroH x2)	-2.938	Asp174<br>(salt)<br>Lys176<br>(H)
Bosutinib	**-2.577**	**Trp113**<br>**(pi-pi)**<br>**Arg171**<br>**(pi-cation x2)**<br>Gly146<br>(aroH)<br>Asp169<br>(H)	-3.582	Tyr123<br>(pi-pi)
Furomazine	-3.649	Asp138<br>(AroH)<br>Lys145<br>(H)<br>Gly146<br>(H)	-3.147	**Cys175**<br>**(H)**<br>Met162<br>(H)
Ibacitabine	-3.832	Asp174<br>(H x2)	-3.678	**Cys175**<br>**(H)**<br>Cys176<br>(H)
Idoxuridine	**-3.761**	**Trp113**<br>**(pi-pi)**<br>**Arg171**<br>**(pi-cation)**<br>Leu111<br>(H)	-3.224	**Cys175**<br>**(H)**<br>Asp174<br>(H)<br>Lys176<br>(pi-cation)
IGS-2,7	**-2.196**	Lys145<br>(aroH)<br>**Arg171**<br>**(Hx2, salt)**	-1.353	Asp174<br>(aroH)<br>Lys176<br>(H and halogen)
KPT 335(verdinexor)	**-3.107**	**Arg171**<br>**(pi-pi)**<br>Asp169<br>(aroH)	-1.887	Met162<br>(H,aroH)
LND-0130436	**-2.394**		-2.572	

		**Gly110**<br>**(H bond)**<br>Gly146<br>(aromatic H bond)		Cys176<br>(halogen)<br>Asp174<br>(H)<br>**Cys175**<br>**(H)**
Olomoucine	**-2.38**	**Gly110**<br>**(pi-pi)**<br>**Trp113**<br>**(aroH)**<br>Asp174<br>(H)<br>Leu111<br>(aroH x2)	-2.772	Arg165<br>(H)<br>Met167<br>(H x2)<br>Asp119<br>(aroH x2)
Panipenam	-4.48	Lys145<br>(H)<br>Lys146<br>(H,aroH)<br>Asp174<br>(H)	-5.615	Hie166<br>(H)<br>Met167<br>(H)<br>Glu122<br>(H, salt)<br>Asp119<br>(H, salt)
PHA767491	**-4.621**	**Trp113**<br>**(pi-pi)**<br>Leu111<br>(AroH)<br>Gly146<br>(AroH)<br>**Arg171**<br>**(pi-cation)**<br>**Gly110**<br>**(H)**	-3.761	Met162<br>(H)
Pioglitazone	**-4.057**	**Trp113**<br>**(pi-pi)**<br>**Gly110**<br>**(H)**<br>**Arg171**<br>**(pi-cation)**<br>Leu111<br>(aroH x2)	-1.202	**Cys175**<br>**(H)**<br>Asp174<br>(aroH, H and salt)
SB 415286	**-3.591**	**Trp113**<br>**(aroH)**<br>**Arg171**<br>**(salt)**<br>**Gly110**<br>**(H)**<br>Asp174<br>(H, aroH)<br>Lys176<br>(halogen)	-3.85	-
SB216763	**-3.536**	**Gly110**<br>**(H)**	-2.92	Lys176<br>(pi-cation)<br>**Cys175**

		Lys145<br>(aroH)<br>Gly146<br>(aroH)<br>**Arg171**<br>**(pi-pi)**<br>Leu111<br>(aroH)		**(aroH, halogen)**<br>Met162<br>(aroH)
TDP-43	**-4.742**	Phe147<br>(pi-pi)<br>Cys145<br>(aroH)<br>**Trp113**<br>**(pi-pi)**<br>**Arg171**<br>**(pi-cation)**<br>Gly146<br>(aroH)<br>Leu111<br>(aroH, H)	-4.127	Arg165<br>(pi-cation)<br>Asp174<br>(aroH x2 and H)<br>Met162<br>(H, aroH)
Tropapride	**-2.795**	Hie143<br>(pi-pi)<br>**Trp113**<br>**(pi-pi x2)**	-3.211	Met162<br>(H)<br>Asp174<br>(H, aroH and salt)
Vorinostat	**-1.138**	Lys176<br>(H)<br>Asp174<br>(H)<br>**Arg171**<br>**(pi-cation)**<br>Gly146<br>(aro H)<br>**Trp113**<br>**(pi-pi)**<br>Leu111<br>(aroH)	-0.05	Met162<br>(H and aroH)<br>Arg165<br>(H)
**Inactive (or decoys)**				
4-carbamoyl-2'-hydroxyiminomethyl-1,1'-oxidimethylenedi(pyridinium) dichloride	-2.998	Lys176<br>(salt)<br>Gly146<br>(aroH)<br>Leu111<br>(H, aroH)	-3.576	Glu122<br>(H)<br>Asp119<br>(H,aroH)
Amphotericin B	-	-	-	-
Clometacin	**-4.328**	**Trp113**<br>**(pi-pi)**<br>**Gly110**<br>**(H)**<br>Gly146<br>(aroH)<br>**Arg171**	-2.567	Lys176<br>(H, salt)<br>Asp174<br>(aroH)<br>Arg165<br>(halogen)

		**(H, salt)**		
Cloxypendyl	-2.956	Phe147 (pi-pi) Arg165 (H x2) Trp172 (H)	-3.175	Gln182 (halogen) Met162 (H) Asp174 (H)
Fipronil	-3.294	No interact	-2.867	Arg165 (H) Asp174 (H)
Flumedroxone-17-acetate	-3.551	Gly170 (H) Lys114 (H)	-2.758	Arg165 (H) Lys176 (H)
Fluperamide	**-3.551**	Phe147 (pi-pi) Gly146 (H, aroH) Leu111 (H) **Trp113 (H) Arg171 (pi-cation)**	-1.869	Arg165 (aroH)
Maduramicin	**-3.5**	Asp169 (H) **Arg171 (H x2,salt) Gly110 (H)**	-	-
Metibride	**-2.739**	Phe147 (pi-pi) **Arg171 (H,pi-cation)** Gly146 (aroH) Leu111 (aroH)	-3.113	**Cys175 (aroH x2)** Arg165 (H x2) Met162 (H,aroH)
Mizolastine	**-3.173**	**Trp113 (pi-pi x3)** Leu111 (aroH) **Arg171 (pi-cation x2)**	-2.546	Asp174 (aroH, H,salt)
Nelzarabine	-3.392	Asp174	-4.609	Met162

		(H)<br>Lys176<br>(H)		(H)<br>Ser163<br>(aroH)<br>**Cys175**<br>**(H)**<br>Asp174<br>(H)
Oxazafone	**-3.846**	Arg165<br>(H)<br>Trp172<br>(H)<br>**Gly110**<br>**(H)**	-4.408	Glu122<br>(aroH)<br>Asp119<br>(H,salt)<br>Asp169<br>(H x2)
Parconazole	**-2.636**	Phe147<br>(aroH)<br>Gly146<br>(aroH)<br>Leu111<br>(aroH)<br>**Arg171**<br>**(pi-cation)**<br>**Gly110**<br>**(H)**	-2.445	Thr126<br>(H)
Perfomedil	**-3.100**	**Trp113**<br>**(pi-pi, H)**<br>Ser144<br>(aroH)	-3.775	Met167<br>(H x2)
Pipobroman	-2.946	No interact	-2.919	Asp169(H)
Pirifibrate	**-3.45**	**Arg171**<br>**(pi-cation)**<br>**Trp113**<br>**(pi-pi)**<br>Leu111<br>(aroH x2)<br>Gly146<br>(aroH)<br>Asp174<br>(H,aroH)	-3.807	Asp119<br>(H)<br>Asp169<br>(H)
Pivoxazepam	**-3.151**	**Gly110**<br>**(H)**<br>**Arg171**<br>**(pi-cation)**<br>**Trp113**<br>**(pi-pi)**<br>Gly146<br>(H)<br>Leu111<br>(aroH x2)	-3.08	**Cys175**<br>**(H x2)**<br>Met162<br>(H)<br>Lys176<br>(pi-cation x2)
Sertindole	-3.663	Phe147<br>(pi-pi)	-3.799	Asp169<br>(H)

				Trp172<br>(pi-pi)<br>Arg165<br>(aroH)
Temelastine	**-3.876**	Lys176<br>(H)<br>**Gly110**<br>**(H)**<br>Lys145<br>(H)<br>**Trp113**<br>**(pi-pi)**	-2.905	Arg165<br>(H)<br>Asp174<br>(H x2)
Thioinosine	**-4.086**	**Arg171**<br>**(H)**<br>Trp172<br>(H)<br>Asp174<br>(H)<br>Lys145<br>(H)	-4.38	**Cys175**<br>**(H x2)**<br>Asp174<br>(H, aroH) Met162<br>(H)
Tiaramide	**-3.326**	**Trp113**<br>**(pi-pi x2)**<br>**Arg171**<br>**(pi-cation x2, H)**<br>Leu111<br>(aroH)<br>Asp174<br>(H)	-3.544	Asp169<br>(H)<br>Asp119<br>(salt,H)<br>Glu222<br>(salt)<br>Hie166<br>(salt)
Tilorone	**-2.643**	**Trp113**<br>**(pi-pi x2)**<br>**Arg171**<br>**(pi-cation)**<br>Leu111<br>(aroH)<br>Gly146<br>(aroH)<br>Lys145<br>(H)	-2.709	Asp174<br>(H,aroH,salt)<br>**Cys175**<br>**(aroH)**<br>Cys176<br>(pi-pi)<br>Met162<br>(aroH)
Uldazepam	-3.043	No interac	-2.879	Tyr123<br>(pi-pi)<br>Asp119<br>(aroH x2)
Vinconate	**-1.865**	**Arg171**<br>**(pi-cation)**	-3.165	Met162<br>(H)

H: H bond interaction; aroH: aromatic H bond; pi-pi interaction; pi-C+: pi- cation interaction; salt: salt bridge interaction; halo: halogen bond.
